# Effect of Macro Fibers on Chloride Permeability and Damage of Concrete Under Uniaxial Compression

**DOI:** 10.3390/ma18040784

**Published:** 2025-02-11

**Authors:** Zengyao Li, Yongqiang Yang, Yihan Wang, Wenqiang Wang, Bailin Zhang

**Affiliations:** 1 Heilongjiang Province Academy of Cold Area Building Research, Harbin 150080, China; 2 Key Laboratory of Earthquake Engineering and Engineering Vibration, Institute of Engineering Mechanics, China Earthquake Administration, Harbin 150080, China; yangyongqiang@iem.ac.cn; 3 Key Laboratory of Earthquake Disaster Mitigation, Ministry of Emergency Management, Harbin 150080, China; 4 School of Municipal and Environment Engineering, Jilin Jianzhu University, Changchun 130118, China; zhuyifan@student.jlju.edu.cn; 5 Key Lab of Structures Dynamic Behavior and Control of Ministry of Education, Harbin Institute of Technology, Harbin 150090, China; 21sk33343@stu.hit.edu.cn (W.W.); 20sk33212@stu.hit.edu.cn (B.Z.); 6 School of Civil Engineering, Harbin Institute of Technology, Harbin 150090, China

**Keywords:** macro fiber, compressive stress level, threshold, chloride diffusion coefficient, ultrasonic testing

## Abstract

In many offshore structures, structural components are often subjected to compressive forces and seawater corrosion. Therefore, understanding their resistance to chloride ion-induced corrosion under compression is crucial. This study investigates the effects of macro polypropylene fibers and macro steel fibers on the chloride permeability and damage of concrete under uniaxial compression. Ultrasonic testing is performed before and after the uniaxial compression test to assess the damage to concrete specimens at different stress levels. Simultaneously, the Rapid Chloride Migration test is conducted on the specimens subjected to various compressive stress levels. The results reveal that the chloride permeability of concrete is influenced by the stress level after uniaxial compression. Additionally, a threshold phenomenon is observed in the chloride permeability: after reaching the threshold stress level, the chloride diffusion coefficient increases significantly. Compared with plain concrete, incorporating macro fibers raises the threshold stress level for chloride ion penetration. Furthermore, this threshold stress level increases with higher fiber content. The variation in ultrasonic velocity with stress level is also found to be an effective indicator for evaluating the chloride permeability of concrete under uniaxial compression. Moreover, a prediction model for the chloride permeability of FRC (fiber reinforced concrete) is proposed based on the results.

## 1. Introduction

The durability of concrete is a critical issue for all concrete structures, particularly for offshore structures exposed to chloride-ion environments. However, the durability of concrete involves multiple factors, and the influencing elements and damage mechanisms are complex. Previous investigations have shown that most of concrete material deterioration occurs under the influence of water and the intrusion of harmful liquids or gases [1]. Therefore, improving the impermeability of concrete is crucial for enhancing its durability.

Anti-chloride ion corrosion is a crucial aspect of durability studies for concrete structures, particularly offshore structures. For offshore structures such as bridge piles, concrete is often subjected to both compression and seawater corrosion [2]. Additionally, certain precast concrete segmental tunnels may primarily endure compressive stresses while simultaneously being subjected to corrosion from the surrounding soil [3]. Therefore, it is crucial to study its ability to resist chloride ion corrosion under pressure. However, current research on chloride ion permeability in concrete has mainly focused on intact specimens under conditions of relatively low compressive stress and in the absence of crack formation [4]. When cracks develop in concrete structures, the chloride ion permeability becomes predominantly influenced by crack characteristics, including crack width and geometry, rather than the density of the matrix material [5,6]. The presence of cracks could accelerate the intrusion of harmful substances, such as chlorides and CO_2_, which can deteriorate the RC structure and seriously impact infrastructure safety.

Given the significant influence of cracks on permeability, researchers have conducted a series of experimental investigations to examine the permeability characteristics of concrete under both tensile and compressive stress conditions [1,7]. Recent studies have shown increasing attention towards concrete permeability under compressive stress, particularly due to its critical implications for the corrosion of offshore structures and concrete segmental tunnels. Firstly, since harmful ions predominantly infiltrate concrete through water or gas transport mechanisms, the water and gas permeability of concrete under compressive stress were investigated. Hoseini et al. [8,9,10,11,12] studied the relationship between compressive stress levels and the water permeability of plain concrete (PC). The results show that permeability is influenced by applied loading, and a permeability threshold phenomenon is also observed. Building on previous research, Choinska et al. [13] investigated the influence of temperature on the permeability of concrete under varying pressure levels. The experimental results indicated that the permeability of specimens increased rapidly when the stress level exceeded 80%. Additionally, Picandet et al. [14] performed gas permeability tests on high-performance steel fiber-reinforced concrete by subjecting cylindrical specimens to compressive loads ranging from 60% to 90% of their compressive strength. The results also showed that the gas permeability of concrete increases gradually with increasing stress levels and increases substantially once the applied load exceeds 90% of the ultimate load.

Building on the aforementioned findings, further experiments were carried out to examine the chloride permeability of concrete under compressive stress. The chloride permeability of concrete can be evaluated using either the Rapid Chloride Permeability Test (RCPT) in accordance with ASTM C1202 [15] or the Rapid Chloride Migration (RCM) method as specified in GB 50082 [16]. The studies revealed that cracks induced by compressive loading are a critical parameter affecting the chloride permeability resistance of concrete. Lim et al. [17] measured the internal micro-crack length of concrete specimens subjected to low uniaxial compressive stress and used the RCPT method according to ASTM C1202 to investigate the chloride permeability characteristics of concrete at different compression levels. It is found that the area and length of microcracks in concrete increase as the level of uniaxial compression load rises, with a threshold effect of uniaxial compressive load on chloride ion permeability. When the axial pressure threshold is exceeded, the cracks link together, leading to a significant increase in the chloride diffusion coefficient of the concrete. In the studies conducted by Samaha and Hover [18], neutron radiography was employed to document microcrack formation under applied loading conditions. The RCPT was utilized to investigate chloride permeability in concrete subjected to uniaxial compressive stress. Their research similarly identified a threshold phenomenon in chloride ion permeability under compressive loading. The results demonstrated that when the uniaxial compressive stress level surpasses 0.75 (relative to the compressive strength), the electrical current passing through the concrete increases by more than 20%. Conversely, when the stress level remains below this threshold, no significant change in electrical Indication was observed. Additionally, Wang et al. [19] investigate the effect of concrete crack parameters (such as crack density, direction, curvature, and width) on the chloride diffusion coefficient under uniaxial compression using the RCM method. The results show that crack width is the most critical factor affecting the chloride diffusion coefficient. Based on this, a relationship between effective crack density, effective crack width, and the chloride diffusion coefficient is proposed. Furthermore, based on the investigation by Saito et al. [20], both monotonic and cyclic uniaxial compressive loading significantly increase chloride permeability when the applied stress exceeds 90% of the compressive strength. Then, Russo et al. [21,22] compared the chloride ion coefficient of cracked and uncracked concrete with different w/c ratios. It was found that the chloride diffusion coefficient of cracked concrete with a 10–75 μm crack width and 5–45 mm crack depth is significantly larger than that of uncracked concrete. Furthermore, with the decrease in the water–cement ratio, the cracked concrete shows a more significant increase in the chloride diffusion coefficient than uncracked concrete. Moreover, recognizing that dry–wet cycles in simulated marine environments can accelerate chloride-induced corrosion, Wang et al. [23] investigated the surface chloride ion concentration and chloride diffusion coefficient of concrete specimens exposed to varying stress levels under cyclic dry–wet conditions. It is found that the stress level threshold for both the surface chloride ion content and the chloride ion diffusion coefficient is 0.3. Based on these findings, an empirical model was proposed to represent the effect of applied stress level and cycle time on chloride ion permeability. In addition to monotonic and cyclic compressive loading, sustained loading must also be considered due to its significant influence on crack parameters. Zhou et al. [24] conducted an investigation into the effect of fly ash content on the chloride diffusion coefficient of concrete under different sustained axial compressive loads. It is found that when the fly ash content is less than 30%, the chloride diffusion coefficient would be significantly influenced by the fly ash content. The chloride diffusion coefficient of concrete first decreases and then increases with the increase in compressive stress level, following a quadratic parabolic relationship with the stress level.

Based on the aforementioned investigations, a distinct stress threshold governing chloride permeability behavior has been identified. This threshold is significantly influenced by crack formation and propagation under compressive loading. For plain concrete (PC), the propagation lacks a restriction effect under compressive. Meanwhile, macro fibers have been shown to improve crack morphology, restrict crack propagation, and enhance toughness in the concrete matrix, as demonstrated in previous investigations [25]. Therefore, macro fibers can effectively reduce chloride permeability in cracked concrete by increasing crack tortuosity and restricting crack width [26]. Currently, the influence of macro fibers on the chloride permeability of unloaded concrete has been extensively investigated in numerous research studies [27]. In these investigations, macro fibers show a slight increase in the chloride permeability of concrete without load. However, there are relatively few studies on the effect of macro fibers on the chloride permeability of concrete under uniaxial compression load. Additionally, the reflection of cracks and damage within the concrete matrix in the aforementioned studies is typically based on results from Scanning Electron Microscopy (SEM) and Mercury Intrusion Porosimetry (MIP), which require cutting specimens into small samples [28,29,30]. There is a lack of non-destructive methods to assess damage and cracks in concrete. Therefore, as a widely applied non-destructive testing method, ultrasonic velocity testing is commonly used for structural durability assessments and building inspections [31,32]. The cracks and damage within the concrete matrix can be detected by comparing the ultrasonic velocity of concrete under different levels of compressive stress.

This investigation is unique because it is carried out via experimental investigations on chloride diffusion properties for steel fiber reinforced concrete (SFRC) and polypropylene fiber reinforced concrete (PFRC) under different compressive stress levels. They are linked to the non-destructive test method of ultrasonic velocity testing. The effect of macro fibers on the threshold of chloride permeability for concrete is clarified through RCM tests following the compression tests. The damage to the fiber-reinforced concrete (FRC) matrix is reflected by changes in ultrasonic velocity under different stress levels [19,33]. A mathematical model to describe the relationship between chloride diffusion coefficient and stress level is established based on the test result. It is able to quickly predict the chloride diffusion coefficient under different stress levels and fiber content.

## 2. Materials and Methods

### 2.1. Materials and Specimens

**Figure 1:**
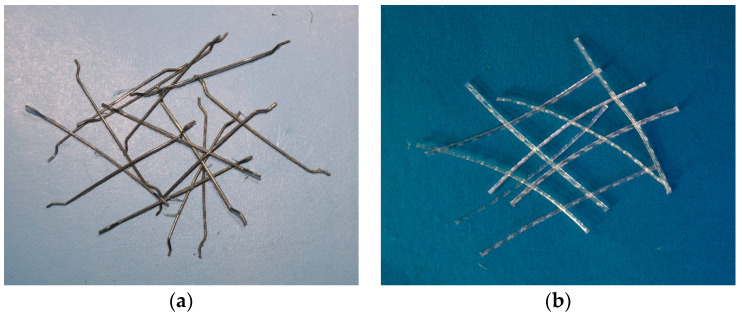
Geometry of fibers: (**a**) macro steel fiber; (**b**) macro PP fiber.

In this study, Ordinary Portland Cement (OPC) compliant with the CEM Ⅰ standard [34] was used. The cement, produced by Dalian Onoda Cement Company (Dalian, China), has a strength grade of 42.5 R, meeting the specified requirements [34]. The fine aggregate is quartz sand with a fineness modulus of 2.51 and a particle size of 0–5 mm, according to GB 14684 [35]. The maximum size of the coarse aggregate is 16 mm as the requirement of GB 14685 [36]. The cement mortar is mixed with the polycarboxylate superplastic agent (SP). The control concrete mix proportion is shown in Table 1. The mixture in Table 1 is specifically designed for a precast fiber-reinforced concrete tunnel engineering project. This study used end-hooked macro steel fibers (SF) from Tangshan, China and straight macro polypropylene fibers (PP) from Ningbo, China. The properties and parameters of the fibers are shown in Figure 1 and Table 2, which are provided by the producers. The fiber contents added to the mixture are 20 kg/m^3^, 30 kg/m^3^, and 40 kg/m^3^ for steel fiber and 4 kg/m^3^, 6 kg/m^3^, and 8 kg/m^3^ for PP fibers.

As per ASTM C39 [37], the fine aggregate, coarse aggregate, cement, water, and admixture are mixed for 3 min. Then, all fibers are gradually added to the mixture. To prevent fiber clumping, the fibers are carefully dispersed and evenly distributed into the concrete matrix, mixed for another 3 min, and then molded within 3 min and covered with cling film. The specimen is de-molded after 24 h and cured in a standard curing room (20 ± 5 °C, 90% humidity) for 28 days.

In this investigation, three concrete cubes 150 mm in size were cast to determine the compressive strength of each kind of concrete. Furthermore, nine concrete cylinders measuring 100 mm in diameter and 200 mm in height were cast for compressive testing for each fiber dosage. Of these, three cylinders were specifically designated to determine the peak stress. After compression, the specimens were cut to perform the RCM test. Table 3 compares the cubic compressive strength of FRC and PC. As shown in Table 3, no significant trend is observed regarding the influence of macro fiber addition at varying contents on the 28-day compressive strength of concrete. This is because the macro fibers show improvement mainly in the post-peak behavior of concrete.

### 2.2. Uniaxial Compression Test

**Figure 2:**
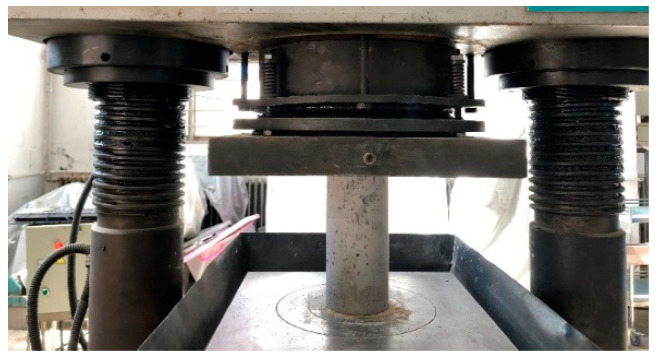
Concrete cylinder under compression test.

Uniaxial compressive tests of concrete cylindrical specimens are performed using a computer-controlled servo universal testing machine located in Dalian, China, which is manufactured by Jinan Shijin company in China, according to ASTM C39 [37]. Figure 2 shows the setup for the compressive test. Before applying different compressive stress levels, the peak stress of the concrete cylinders needs to be determined. Three cylinders for each fiber dosage are tested. The stress level is determined based on the average peak stress for each concrete mix. The loading rate for the compressive test is set at 0.3 MPa/s. After reaching the specified stress level, the compressive load is held for 30 min before unloading. Six specimens are tested for each stress level. Although it is uncommon to load structures beyond approximately 50% of their capacity in engineering practice because of safety factors, it is essential to investigate higher stress levels to gain a comprehensive understanding of chloride permeability behavior. Therefore, compressive stress levels ranging from 0.2 to 0.9, with intervals of 0.1, were selected for investigation.

### 2.3. Ultrasonic Pulse Velocity Through Concrete

**Figure 3:**
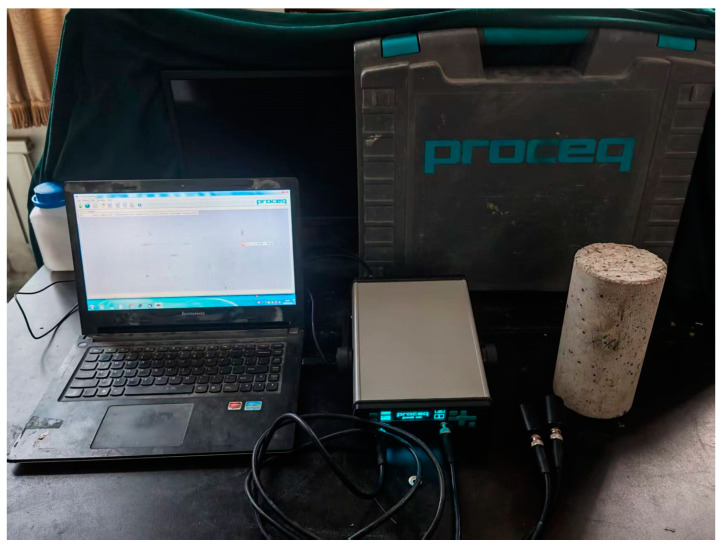
Ultrasonic detector of concrete.

Ultrasonic waves propagate at different speeds through different matrices, allowing them to be used to detect damage within concrete. In this study, the Pundit Lab ultrasonic detector from Switzerland and located in Dalian, China, shown in Figure 3, is used to measure ultrasonic wave velocity, according to ASTM C597 [38]. The transmit and receive frequencies are set to 54 kHz, with a data acquisition period of 0.4 μs. The ultrasonic wave velocity inside the concrete cylinder specimens is measured both before and after the uniaxial compression test. The relationship between the ultrasonic wave velocity and the damage variables of each group of specimens under different stress levels is then analyzed.

The specimens are placed on foam boards to isolate external influences, with the poured side facing upward. Before testing, the apparatus is tuned and calibrated. The probes at both ends of the instrument are coated with a small amount of petroleum jelly as a coupling agent and placed against the center of the two square surfaces of the concrete specimen to begin the measurement. Each specimen is measured three times, and the average of the three measurements, provided the errors are within 5% of the test value, is taken as the wave velocity for that specimen. The final ultrasonic velocity test value for each specimen is the average of the three individual test values.

Equation (1) shows the relationship between ultrasonic velocity and the dynamic elastic modulus of concrete. Additionally, Equation (2) presents the calculation of the damage parameter. By combining Equations (1) and (2), the damage parameter can be calculated using Equation (3).

(1) $$v^{2}=\frac{E}{\rho }\cdot \frac{1-\mu }{\rho \left(1+\mu \right)\left(1-2\mu \right)}$$

where *v* is the ultrasonic velocity of the specimen (m/s); *ρ* is the density of the concrete (kg/m^3^); *μ* is the Poisson’s ratio of the material; and *E* is the dynamic elastic modulus (MPa).

(2) $$D_{d}=1-\left(\frac{E_{n}}{E_{0}}\right)$$

(3) $$D_{d}=1-\left(\frac{v_{n}^{2}}{v_{0}^{2}}\right)$$

where *v_n_* is the ultrasonic velocity test value of the specimen after axial compression (m/s); *v*_0_ is the ultrasonic velocity test value of the specimen before loading (m/s); *D*_*d*_ is the internal damage variable of the specimen after axial compression (%); *E* is the dynamic elastic modulus (MPa); and *E*_0_ is the dynamic elastic modulus of the specimen before loading (MPa).

### 2.4. RCM Test

**Figure 4:**
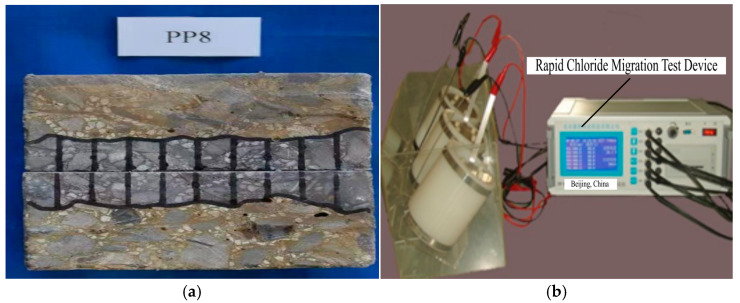
Test setup of RCM: (**a**) specimen color outline; (**b**) RCM device.

After the compressive and ultrasonic tests, the specimen is cut to a depth of 50 mm to perform the RCM test [16]. The chloride diffusion coefficient of concrete under different uniaxial compression stress levels is tested using the NTB-DAL chloride diffusion coefficient tester manufactured by NEL-Der in Beijing, China. After the test, the specimen is split along its depth and immediately sprayed with a 0.1 mol/L AgNO_3_ solution for 15 min. Then, the color line is outlined, and the section is divided into ten equal parts. The penetration depth is measured at various points, and the average penetration depth of the 7 middle points is recorded with an accuracy of 0.1 mm. For each group of test blocks, six tests are performed, and the average of all test blocks is used to calculate the chloride penetration coefficient. The chloride diffusion coefficient is calculated as shown in Formula (4). The test setup is shown in Figure 4.

(4) $$D_{RCM}=\frac{0.0239\times \left(273+T\right)L}{\left(U-2\right)t}\left(X_{d}-0.0238\sqrt{\frac{\left(273+T\right)LX_{d}}{U-2}}\right)$$

where *D_RCM_* is the unsteady chloride migration coefficient of the concrete specimen (m^2^/s); *U* is the test voltage (V); *T* is the average temperature of the anodic solution (°C); *L* is the thickness of the specimen (mm); *X_d_* is the average value of chloride ion penetration depth; and *t* is the test time (h).

## 3. Results and Discussion

### 3.1. Chloride Diffusion Coefficient of Concrete Under Compression

**Figure 5:**
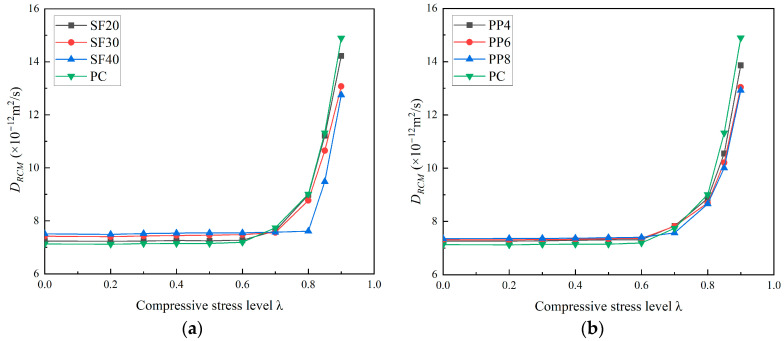
Relationship between chloride diffusion coefficient and uniaxial compressive stress level of FRC. (**a**) SFRC; (**b**) PFRC.

The variation curves of the chloride diffusion coefficients for PC, PFRC, and SFRC specimens, with respect to the level of uniaxial compressive load, are shown in Figure 5. The trend in chloride diffusion coefficients at different stress levels is similar for each group of specimens (Figure 5a,b). It can be seen from Figure 5 that the chloride diffusion coefficient increases with the increasing stress level. Compared with PC, FRC shows a slightly higher chloride diffusion coefficient. This is because, at relatively low stress levels, the chloride diffusion coefficient depends on the pore structure of the concrete matrix. The addition of macro fibers into the concrete would increase the interface between fiber and concrete matrix. Thus, a slight increase in chloride diffusion coefficient would be observed due to the increase in pores. Additionally, a threshold value is observed based on the uniaxial compression stress level. Beyond this threshold, the chloride diffusion coefficient increases significantly due to crack propagation. The threshold value, however, varies with fiber dosage. For SF20, SF30, and SF40 specimens, the axial compression threshold stress levels are 0.6, 0.7, and 0.8, respectively. For PP4, PP6, and PP8 specimens, the threshold stress levels are 0.6, 0.6, and 0.7, respectively. It can be observed that steel fibers have a greater impact on the threshold compared to polypropylene fibers. At these stress levels, the chloride diffusion coefficients of PC become higher than FRC specimens.

For the PC, SF20, PP4, and PP6 specimens, the D_RCM_ values show little change below the threshold value of 0.6. At λ = 0.8, the D_RCM_ values are 9.01 × 10^−12^ m^2^/s, 8.98 × 10^−12^ m^2^/s, 8.86 × 10^−12^ m^2^/s, and 8.69 × 10^−12^ m^2^/s, which represent increases of 26.37%, 24.03%, 21.2%, and 18.07% compared to the values at λ = 0.6, respectively. For the SF30 and PP8 specimens, the D_RCM_ values remain nearly unchanged below the threshold value of 0.7. When λ = 0.8, the D_RCM_ values are 8.77 × 10^−12^ m^2^/s and 8.66 × 10^−12^ m^2^/s, indicating increases of 16.01% and 14.4% compared to the values at λ = 0.6, respectively. At λ = 0.9, the D_RCM_ values rise to 13.07 × 10^−12^ m^2^/s and 12.92 × 10^−12^ m^2^/s, showing increases of 72.88% and 70.67%, respectively, compared to the values at λ = 0.6. For the SF40 specimens, the D_RCM_ values remain almost constant below the threshold value of 0.8. At λ = 0.85, the D_RCM_ is 9.48 × 10^−12^ m^2^/s, representing a 24.57% increase compared to the value at λ = 0.6. When λ = 0.9, the D_RCM_ values increase to 12.75 × 10^−12^ m^2^/s and 12.92 × 10^−12^ m^2^/s, respectively, showing a 67.54% increase compared to the value at λ = 0.6. Based on the analysis, SFRC and PFRC exhibit more distinct thresholds for uniaxial compression compared to PC. This suggests that SFRC and PFRC have lower chloride permeability coefficients at the same uniaxial compression stress level once the threshold is exceeded. In other words, macro fibers help reduce the rate of increase in chloride permeability after the threshold load is surpassed. Moreover, the growth rate of chloride permeability decreases significantly with increasing fiber content. The higher threshold values also indicate the effective role of macro fibers in restricting crack propagation.

### 3.2. Effect of Macro Fibers on Internal Damage of Concrete Under Compression

**Figure 6:**
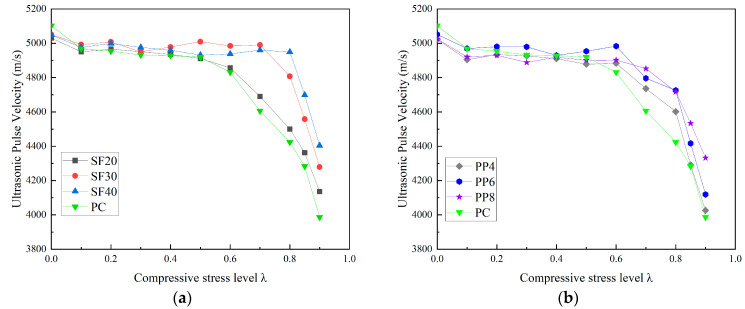
Relationship between ultrasonic pulse velocity and stress level of concrete subjected to uniaxial compressive load. (**a**) SFRC; (**b**) PFRC.

The relationship between ultrasonic velocity values and fiber-reinforced concrete at different levels of uniaxial compressive load is shown in Figure 6. A similar threshold trend, as observed with the chloride diffusion coefficient, is also illustrated for the ultrasonic velocity. Additionally, the threshold value increases progressively with the increase in fiber content. Figure 6a demonstrates that the uniaxial compressive threshold stress levels for PC, SF20, SF30, and SF40 specimens are 0.6, 0.6, 0.7, and 0.8, respectively. The SF20 specimen shows almost no change in threshold value compared to the PC specimen, likely due to the lower steel fiber dosage. The inclusion of steel fibers reduces the rate of ultrasonic velocity once the threshold value is exceeded. The ultrasonic velocities corresponding to the uniaxial compressive threshold stress levels of PC, SF20, SF30, and SF40 specimens are 4831 m/s, 4856 m/s, 4990 m/s, and 4949 m/s, respectively. As shown in Figure 6b, both macro steel fibers and polypropylene fibers help mitigate the reduction in ultrasonic velocity. However, the impact of polypropylene fibers on the concrete threshold and wave velocity reduction is less pronounced than that of steel fibers.

**Figure 7:**
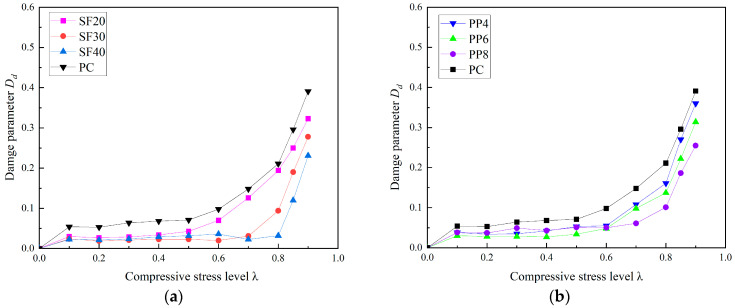
Relationship between damage parameter and stress level of concrete subjected to uniaxial compressive load. (**a**) SFRC; (**b**) PFRC.

The relationship between the damage parameter and the compressive stress level of fiber-reinforced concrete is shown in Figure 7. From Figure 7, it can be observed that the inclusion of macro polypropylene (PP) fibers and macro steel fibers helps to reduce the internal damage of concrete specimens under the same uniaxial compressive stress level. Furthermore, the effect of macro PP fibers on reducing the concrete damage parameter appears to be less significant compared to that of macro steel fibers. This is likely due to the greater restrictive effect of macro steel fibers, which results in a higher threshold stress level, beyond which significant changes in the damage parameter of the specimen occur.

**Figure 8:**
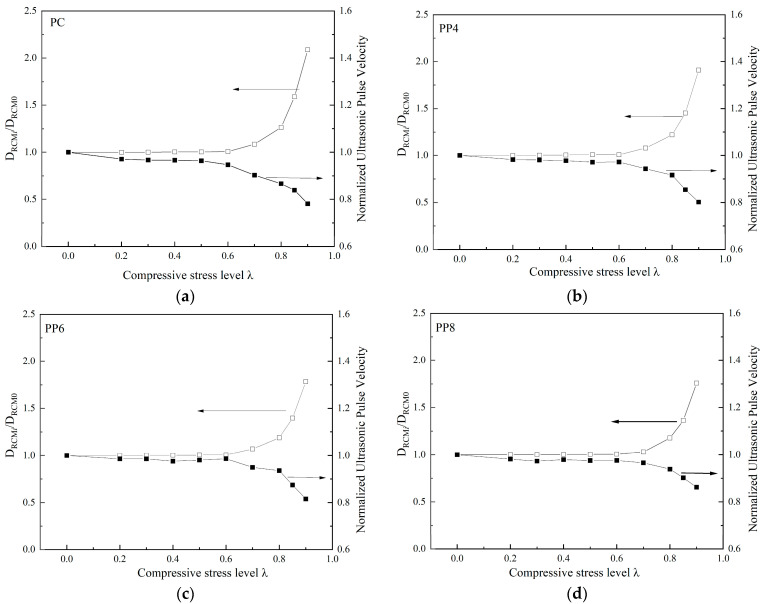
Effect of uniaxial compressive load level on the ultrasonic pulse velocity and chloride diffusion coefficient of specimens: (**a**) PC; (**b**) PP4; (**c**) PP6; (**d**) PP8.

**Figure 9:**
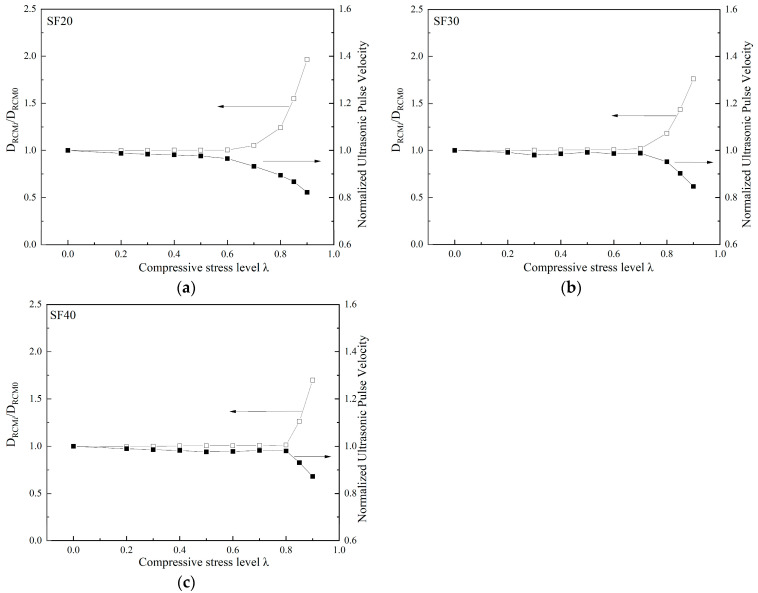
Effect of uniaxial compressive load level on the ultrasonic pulse velocity and chloride diffusion coefficient of specimens: (**a**) SF20; (**b**) SF30; (**c**) SF40.

To prove the correlation between ultrasonic velocity and chloride ion permeability of fiber-reinforced concrete, the chloride diffusion coefficient and ultrasonic velocity measurement value of each group of specimens before loading were taken as the reference values, respectively. The ratio of the permeability coefficient and ultrasonic velocity measurement to the reference value is the vertical axis. Figure 8 compares the normalized ultrasonic velocity and chloride ion diffusion coefficient under different uniaxial compressive stress levels for PFRC. Moreover, Figure 9 shows the comparison between SFRC specimens. The comparison in Figure 8 demonstrates a significant consistency between the changes in ultrasonic velocity and the changes in the chloride diffusion coefficient. Ultrasonic velocity can thus be used to evaluate the chloride ion diffusion performance of concrete under different compressive stress levels. At the same time, the macro fibers play a role in restricting crack propagation, which reduces the damage to the concrete. As a result, both the loss of ultrasonic velocity and the chloride ion diffusion coefficient decrease after the threshold, showing a lower value under the same stress level compared to plain concrete (PC).

**Figure 10:**
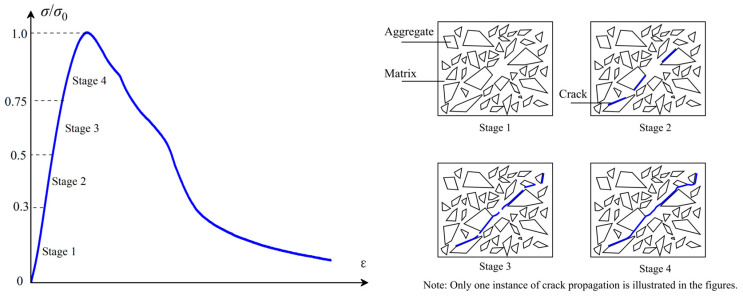
Constitutive Behavior of Concrete under Uniaxial Compression (left blue curve) and Schematic Illustration of Crack Propagation under Uniaxial Compressive Loading.

The ultrasonic velocity and chloride ion diffusion coefficient of concrete under uniaxial compressive loading are closely associated with the damage evolution within the concrete matrix. As shown in Figure 10, damage continues to accumulate during the compression of concrete. The chloride permeability and ultrasonic velocity of concrete under uniaxial compressive loading are influenced by the development of internal cracks. The damage progression before peak stress can be divided into four stages, as outlined in Ref. [28].

In Stage 1, minimal damage occurs, but it does not significantly affect the properties of the concrete. In Stage 2, when the stress level reaches 30–50% of the ultimate (peak) stress of the concrete, microcracks may form in the interfacial transition zone (ITZ) between the aggregate and the cement matrix. As a result, a slight increase in the chloride ion diffusion coefficient is observed. Moreover, macro fibers have little influence on the ultrasonic velocity and chloride ion diffusion coefficient at this stage, as their restraining effect on microcracks is relatively limited. Additionally, the addition of macro fibers increases the volume of the ITZ, which explains the slight increase in the chloride ion diffusion coefficient for FRC specimens compared to plain concrete before reaching the threshold stress level. At this stage, the microcracks in the ITZ remain stable.

In Stage 3, when the stress level is between 50 and 75% of the ultimate stress for plain concrete, cracks from the ITZ begin to propagate into the concrete matrix. The damage threshold typically occurs during this stage. Macro cracks start to form and propagate within the concrete. As a result, the damage parameter experiences a marked increase in both plain concrete and FRC specimens with relatively low fiber content. This leads to a rapid rise in the chloride ion diffusion coefficient, particularly for plain concrete. In FRC specimens, macro fibers begin to exert a significant restraining effect on the propagation of macro cracks. Consequently, the damage parameter shows a relatively lower increase compared to plain concrete, and the chloride ion diffusion coefficient for FRC remains lower than that of plain concrete. The degree of restriction varies depending on the fiber dosage, with different fiber dosages exhibiting varying thresholds in both damage and chloride ion diffusion coefficient for FRC.

In stage 4, macro cracks continue to accumulate, and crack surfaces begin to form and propagate. Damage increases rapidly, as does the chloride ion diffusion coefficient. However, FRC specimens continue to demonstrate lower damage and chloride ion diffusion coefficients due to the bridging effect of the macro fibers.

### 3.3. Data Fitting Analysis of Concrete Chloride Diffusion Coefficient Under Compression

Based on previous investigations, the relationship between the chloride ion diffusion coefficient and the stress level for fiber-reinforced concrete (FRC) is established using an exponential growth model via statistical fitting. The analysis of fiber-reinforced concrete (FRC) under compressive loading reveals that the relationship between chloride diffusion and the stress level initially exhibits a slight change at low stress levels (below 50% of the compressive strength). Given the experimental results, the relationship between chloride permeability and compressive stress level is similar to the exponential growth model. Initially, the chloride permeability coefficient of concrete shows a steady tendency with the increase in compressive stress level. After the turning point, a significant increase could be observed, as demonstrated by the experimental results presented in this study. Equation (5) presents the models based on statistical fitting for steel fiber-reinforced concrete (SFRC) and polypropylene fiber-reinforced concrete (PFRC), respectively. The parameters *A*, *B*, and *t* in Equation (5) are material-dependent.

(5) $$D_{RCM}=A+Be^{\frac{\lambda }{t}}$$

**Figure 11:**
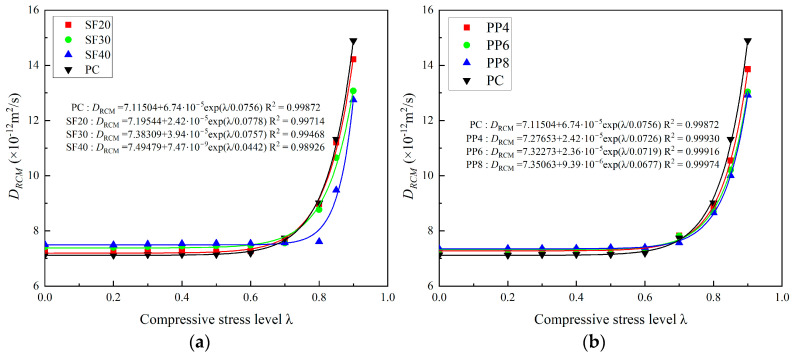
Fitting of the *D*_RCM_ for different types of specimens: (**a**) SFRC and (**b**) PFRC.

Figure 11 shows the comparison of fitting curves and the experimental results of the chloride permeability of SFRC and PFRC. It is evident that the distinct restriction effects of polypropylene fibers and steel fibers on the chloride diffusion coefficient are clearly reflected by the fitted curves in this model. As the fiber dosage increases, the growth of the chloride diffusion coefficient at relatively high levels of compressive stress is limited by the presence of macro fibers, as shown in Figure 11. Furthermore, by comparing these formulas with the experimental results of concrete chloride ion permeability under uniaxial compressive loading from other scholars (Sun [22] and Zhang [23]), the applicability of the proposed model is further validated. This formula describes the rapid calculation of the chloride diffusion coefficient for a given specimen, based on the specified fiber type, content, and uniaxial compressive stress level. Parameter A represents the chloride permeability of concrete without stress applied.

It should be noted that the equations are currently limited to the scope of this study. To fill the sample space, additional related experiments should be conducted in future studies.

## 4. Conclusions

In this paper, the effects of macro fibers on chloride ion resistance and damage parameters under different axial compressive stress levels are compared using concrete axial compression tests, ultrasonic velocimetry tests, and RCM tests. Additionally, the damage parameters and chloride diffusion coefficients are analyzed based on the experimental results. The conclusions drawn from the experimental and analytical findings are as follows:

(1) The chloride ion diffusion coefficient increases with the axial compressive stress level for both plain concrete and fiber-reinforced concrete (FRC). A threshold is observed in the relationship between the chloride ion diffusion coefficient and stress level. After reaching this threshold, a rapid increase in the diffusion coefficient occurs. Compared with plain concrete, FRC exhibits a relatively lower diffusion coefficient after the threshold. Moreover, the threshold for FRC is higher than that for plain concrete. Furthermore, higher fiber content results in a smaller chloride ion diffusion coefficient. As fiber content increases, the uniaxial compression threshold increases in both steel fiber-reinforced concrete (SFRC) and polypropylene fiber-reinforced concrete (PFRC).
(2) Ultrasonic velocimetry tests accurately reflect the damage evolution in concrete under varying stress levels. The formation and propagation of cracks inside concrete under different axial compression stress levels can be detected by changes in ultrasonic velocity. The tests demonstrate that ultrasonic testing is a feasible method for evaluating chloride ion permeability under uniaxial compression.
(3) As indicated by the ultrasonic velocimetry tests, macro fibers effectively restrict the propagation of macro cracks within the concrete matrix, helping to delay damage accumulation. Consequently, the chloride ion diffusion coefficient of FRC is lower than that of plain concrete. Macro fibers improve the anti-chloride ion permeability of concrete, particularly beyond the threshold stress level.
(4) Under axial compression, the content and stress level of macro steel fibers and PP fibers exhibit a similar effect on the chloride diffusion coefficient. Prior to reaching the axial compression threshold, the relationship between the stress level and the chloride diffusion coefficient remains nearly constant. The fiber content shows a decreasing effect on the chloride diffusion coefficient. After the threshold, the relationship rapidly increases. Based on the fitting results, an exponential growth model could be adopted to describe the relationship.

Future research will involve conducting comprehensive statistical analysis with an expanded sample space. Additionally, the predictive capabilities of the model will be improved by integrating various fiber types and exploring a wider spectrum of dosage parameters.

## Figures and Tables

**Table 1:** Mix proportion of concrete (kg/m^3^).

Cement	Gravel	Sand	Water	Superplasticizer	w/b
450	1070	714	180	1.25	0.4

**Table 2:** Performance parameters of fiber.

Type	Shape of Fiber	Length /mm	Diameter /mm	Aspect Ratio	Tensile Strength /MPa	Elastic Modulus /GPa
Macro PP fiber	Straight	40	0.60	67	600	7
Macro steel fiber	Hooked-end	40	0.50	80	1150	200

**Table 3:** Compressive strength (28 d) of concrete specimens.

Code	SF	PP	Compressive Strength /MPa
PC	-	-	54.5 (CV = 0.071)
SF20	20 kg/m^3^	-	58.4 (CV = 0.083)
SF30	30 kg/m^3^	-	61.1 (CV = 0.092)
SF40	40 kg/m^3^	-	59.0 (CV = 0.061)
PP4	-	4 kg/m^3^	55.8 (CV = 0.086)
PP6	-	6 kg/m^3^	58.2 (CV = 0.072)
PP8	-	8 kg/m^3^	57.7 (CV = 0.082)

The “-” symbol indicates that no specific fiber was utilized or identified in the given context.

## Data Availability

The original contributions presented in this study are included in the article. Further inquiries can be directed to the corresponding author.
